# Subclinical Left Atrial Remodeling in Healthy Adults with Left Ventricular ‘Rigid Body Rotation’—Detailed Analysis from the Three-Dimensional Speckle-Tracking Echocardiographic MAGYAR-Healthy Study

**DOI:** 10.3390/jcm13237006

**Published:** 2024-11-21

**Authors:** Attila Nemes

**Affiliations:** Department of Medicine, Albert Szent-Györgyi Medical School, University of Szeged, H-6725 Szeged, Hungary; nemes.attila@med.u-szeged.hu; Tel.: +36-62-545220; Fax: +36-62-544568

**Keywords:** left atrial, rigid body rotation, volume, strain, three-dimensional, speckle tracking

## Abstract

**Background.** While the basal region of the left ventricle (LV) rotates in a clockwise (cw) direction, the apical regions of the LV rotate in a counterclockwise (ccw) direction in healthy circumstances. Although LV rotational mechanics help optimize LV ejection, in some cases, LV twist is missing. This clinical situation, when the LV base and the apex rotate in the same cw or ccw direction, is called LV ‘rigid body rotation’ (LV-RBR). Three-dimensional speckle-tracking echocardiography (3DSTE) seems to be optimal for the simultaneous assessment of the LV and the left atrium (LA). Therefore, the present study aimed to determine the features of LA remodeling in healthy adults having 3DSTE-derived LV-RBR as compared to subjects with normally directed LV rotational mechanics. **Methods.** This study consisted of 165 healthy subjects (mean age: 33.1 ± 12.3 years, 75 males), from which 156 individuals showed normally directed LV rotational mechanics, while 9 cases had LV-RBR. **Results.** When LV-RBR subjects were compared to subjects with normally directed LV rotational mechanics, all LA volumes were increased with preserved LA stroke volumes and (non-significantly) reduced LA emptying fractions. When subgroups were compared with each other, it has been clarified that an enlargement of the LA with increased volumes was limited only to ccwLV-RBR cases. While reduced global peak LA longitudinal strain could be detected in LV-RBR subjects as compared to subjects with normally directed LV rotational mechanics, which was limited to cases with the ccw form of LV-RBR (15.1 ± 4.7% vs. 26.6 ± 9.0%, *p* < 0.05), the global peak LA radial strain was increased in subjects with cwLV-RBR (−23.4 ± 6.3% vs. −14.7 ± 8.0%, *p* < 0.05). Increased global LA radial strain at atrial contraction could be detected in LV-RBR subjects (−9.9 ± 7.1% vs. −5.2 ± 5.2%, *p* < 0.05), which was present in both ccw and cw LV-RBR cases. **Conclusions.** In healthy adults presenting LV-RBR, subclinical LA remodeling could be detected in both forms of LV-RBR, but more pronounced in those who present a counterclockwise-oriented form.

## 1. Introduction

While the basal region of the left ventricle (LV) rotates in a clockwise direction (cw) in systole, the apical LV region rotates in a counterclockwise (ccw) direction at the same time in healthy circumstances [1,2,3,4,5]. Although LV rotational mechanics have a significant role in the optimalization of the LV ejection, in some cases, LV twist is missing [1,2,3,4,5]. This clinical situation, when the LV base and apex rotate in the same cw or ccw direction is called LV ‘rigid body rotation’ (LV-RBR), which has been demonstrated to be present not only in certain pathologies, but in healthy subjects as well [6,7]. This rare clinical situation was present in approximately 6% of healthy adults with specific segmental abnormalities in LV deformation being present only in subjects with cwLV-RBR [7,8].

In recent years, there is a central role of non-invasive cardiovascular imaging for redefining cardiovascular risk characteristics in seemingly healthy individuals, with particular emphasis on the utility of myocardial strain in this context [9]. Due to the fact, that LV and the left atrium (LA) cooperate closely with each other during the cardiac cycle, and three-dimensional speckle-tracking echocardiography (3DSTE) seems to be optimal for the simultaneous volumetric and functional assessment of the LV and LA [10,11,12,13,14], the present study aimed to determine features of LA remodeling in healthy adults having 3DSTE-derived LV-RBR as compared to subjects with normally directed LV rotational mechanics.

## 2. Methods

The presented findings are part of the ‘Motion Analysis of the heart and Great vessels bY three-dimensionAl speckle-tRacking echocardiography in Healthy subjects’ (MAGYAR-Healthy) Study, which has been organized at the University of Szeged, from the point of view that the authors—among other aims—find correlations between 3DSTE-derived and other parameters (’Magyar’ means ’Hungarian’ in Hungarian language) (starting date: 1 November 2011–ongoing). This investigation was carried out in accordance with the Declaration of Helsinki (as revised in 2013) and the Institutional and Regional Human Biomedical Research Committee (University of Szeged) approved it under the registration number of 71/2011 (and updated versions); and all participants gave informed consent. This retrospective cohort study comprised 165 healthy adults being in sinus rhythm with a mean age of 33.1 ± 12.3 years (75 males). In all subjects, physical examination, electrocardiography (ECG), two-dimensional (2D) Doppler echocardiography, and 3DSTE have been performed with findings being in the normal range in all subjects. None of them had any known pathology or other clinical situation which could affect the findings. None of the cases smoked, used drugs, or had a body mass index larger than 30 kg/m^2^. None of the participants was an athlete or performed yoga regularly. According to definition, 156 cases had normally directed LV rotational mechanics, while 9 subject had LV-RBR [6].

The same 2D Doppler echocardiography equipment (Artida^TM^, Toshiba Medical Systems, Tokyo, Japan) attached to a PST-30BT (1–5 MHz) phased-array transducer was used in all cases. Routine examinations included quantification of LA and LV dimensions, measurement of LV ejection fraction by modified Simpson’s method, exclusion of valvular regurgitation and stenosis and measurement of early (E) and late (A) diastolic mitral inflow velocities (and their ratio) by Doppler echocardiography [15].

The 3DSTE examinations were performed in two steps. As a first step, 3D echocardiographic datasets were acquired using the same Artida^TM^ echocardiography tool attached to a matrix-array PST-25SX transducer having 3D capability with ECG gating following optimalization of the images including magnitude, gain, etc., focusing on the LA and the LV. Acquisitions were performed during 6 cardiac cycles, acquiring a subvolume during each cycle. Subvolumes were stitched together by the software helping to create a full volume looks like a pyramid [11,12,13,14].

As a second step, acquired 3D echocardiographic datasets were used to formate virtual 3D cast of the LA and LV to determine their volumes and rotational parameters respecting the cardiac cycle. For this aim, a special vendor-provided software called 3D Wall Motion Tracking (Ultra Extend, Toshiba Medical Systems, Tokyo, Japan, version 2.7) was used. Measurements were performed offline at a later date. LV/LA-focused images were used to create apical longitudinal 2-chamber (AP2CH) and 4-chamber (AP4CH) views of LV/LA and apical, midventricular and basal cross-sectional LV views, and basal, midatrial, and superior cross-sectional LA views. Following definition of endocardial surface of the LV apex and septal and lateral edges of the LV and mitral annulus (MA), a virtual 3D cast of the LV has been created after a sequential analysis. In the case of the LA, the observer detected the endocardial LA border by setting several markers between the edge of the septum-MA and the edge of the lateral LV wall-MA.

LV rotational parameters provided using the virtual 3D LV cast were the following:

- Basal and apical LV rotations;
- LV twist (net sum of apical and basal LV rotations);
- Time-to-LV twist.

**Figure 1:**
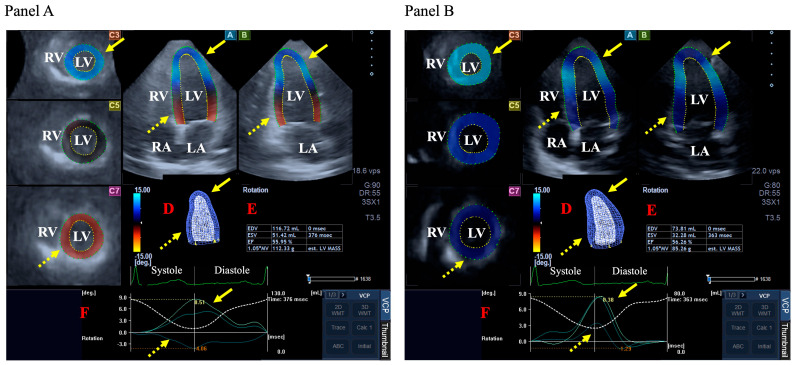
Three-dimensional (3D) speckle-tracking echocardiography-derived assessment of the left ventricle (LV) in a subject with normally directed LV rotational mechanics (**Panel A**) and a case with LV ‘rigid body rotation’ (**Panel B**): apical longitudinal four-chamber (**A**) and two-chamber views (**B**) and basal (**C3**), midventricular (**C5**), and apical (**C7**) short-axis views are shown together with 3D cast of the LV (**D**), volumetric LV parameters, and LV ejection fraction (**E**). Time–LV volume changes (dashed white line) and time–apical (yellow arrow) and basal (dashed yellow arrow) LV rotational curves (**F**) are also presented. According to normal LV physiology, apical LV rotation is counterclockwise-directed, while basal LV rotation is clockwise-directed, as seen in (**Panel A**), while both apical and basal LV rotations are in the same counterclockwise direction demonstrating an absence of LV twist, referred to as LV ‘rigid body rotation’, as seen in (**Panel B**). Abbreviations. LV = left ventricle; LA = left atrium; RA = right atrium; RV = right ventricle.

If LV twist could not be measured, LV-RBR was considered to be present [6]. In cwLV-RBR, basal and apical LV regions rotate in the same cw direction (apical region of the LV rotates in a direction opposite to the normal counterclockwise direction), while in ccwLV-RBR, the LV regions rotate in the same ccw direction (basal region of the LV rotates in a direction opposite to the normal clockwise direction) (Figure 1).

LA volumetric and functional parameters were determined using the virtual 3D LA cast were the following [16]:

- End-systolic maximum LA volume (V_max_, before mitral valve opening);
- Early diastolic LA volume before atrial contraction (V_preA_, at the time of the P-wave on the ECG);
- End-diastolic minimum LA volume (V_min_, before mitral valve closure).

Using LA volumes, the following LA volume-based functional properties have been calculated:

featuring LA reservoir function:

- Total stroke volume (SV) = V_max_ − V_min_;
- Total emptying fraction (EF) = Total SV/V_max_.

featuring LA conduit function:

- Passive SV = V_max_ − V_preA_;
- Passive EF = Passive SV/V_max_.

featuring LA active contraction:

- Active SV = V_preA_ − V_min_;
- Active EF = Active SV/V_preA_.

**Figure 2:**
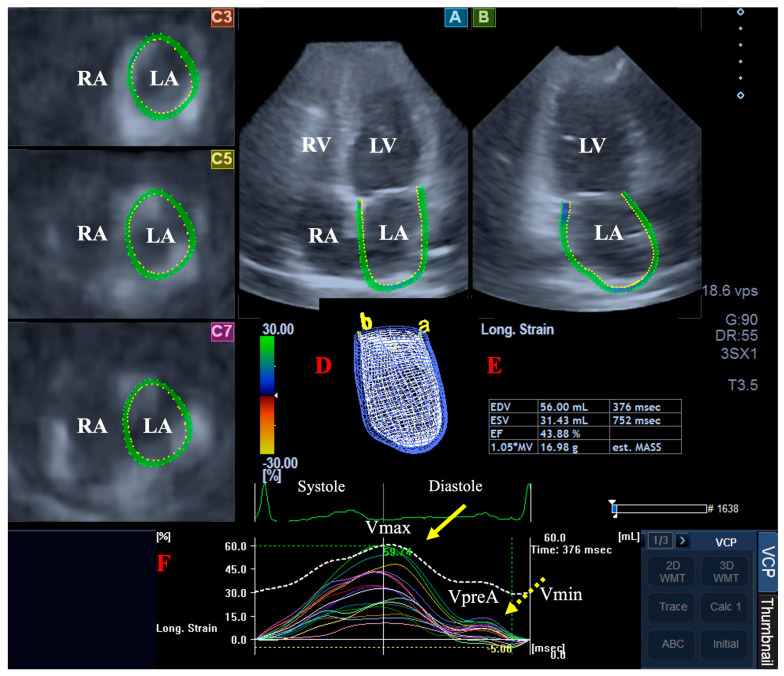
Three-dimensional (3D) speckle-tracking echocardiography-derived assessment of the left atrium (LA) in a healthy subject: apical longitudinal four-chamber (**A**) and two-chamber views (**B**) and superior (**C3**), midatrial (**C5**), and basal (**C7**) short-axis views are shown together with 3D cast of the LA (**D**) and volumetric LA parameters (**E**). Time–LA volume changes (dashed white line) and time–global (white line) and segmental (colored lines) longitudinal peak LA strains (yellow arrow) and LA strains at atrial contraction (dashed yellow arrow) (**F**) are also presented. Abbreviations. LV = left ventricle; LA = left atrium; RA = right atrium; RV = right ventricle; V_max_ = systolic maximum LA volume; V_preA_ = early diastolic pre-atrial contraction LA volume; V_min_ = late diastolic LA volume.

Using the same virtual 3D cast of the LA, several global (featuring the whole LA), segmental, mean segmental, and regional (featuring basal, midatrial, and superior LA regions) strains could be measured as a quantitative feature of contraction/relaxation of the LA wall in radial (radial strain, RS), longitudinal (longitudinal strain, LS) and circumferential (circumferential strain, CS) directions [17]. Two complex LA strains were also measured [3D strain (3DS) as a combination of all linear strains and area strain (AS) as a combination of LS and CS]. Due to the fact that LA strain curves have two peaks, end-systolic reservoir LA function is represented by the first peak, while end-systolic booster pump function is represented by the second peak (LA systole) (Figure 2).

All data are presented as a mean ± standard deviation format for continuous variables and count (percentage) format for categorical variables. *p* less than 0.05 was considered to be statistically significant. To test normality, the Shapiro–Wilk test was used with Student’s *t*-test with Welch correction when the distribution was normal, and Mann–Whitney-Wilcoxon test when distribution proved to be non-normal. The Bonferroni method was used following a one-way ANOVA, where appropriate. For categorical variables, comparisons were performed by Fisher’s exact test. SPSS version 22 (SPSS Inc., Michigan, IL, USA) software was used for statistical analyses.

## 3. Results

Demographic and clinical characteristics. Table 1 presents a comparison of the demographic and clinical features of healthy subjects with normally directed LV rotational mechanics vs. cases with LV-RBR. The population of healthy subjects examined for this manuscript is partly the same as that analyzed in a previous paper [8].

Two-dimensional Doppler echocardiography. None of the routine 2D Doppler echocardiographic parameters differed between cases with normally directed LV rotational mechanics and subjects with LV-RBR and were within the normal reference ranges. However, cwLV-RBR subjects had smaller-sized LA compared to ccwLV-RBR cases and individuals showing normally directed LV rotational mechanics (32.0 ± 1.8 mm vs. 39.1 ± 1.4 mm and 36.7 ± 4.0 mm, *p* < 0.05 and *p* < 0.05, respectively), and other parameters did not differ when comparing cw vs. ccw forms of LV-RBR. None of the individuals showed ≥grade 1 valvular regurgitation or any degree of stenosis.

3DSTE-derived basal and apical LV rotations. In 156 individuals with normally directed LV rotational mechanics, basal LV rotation was −4.26 ± 2.11 degrees (ranging between −0.24 to −11.63 degrees), while apical LV rotation proved to be 9.55 ± 3.53 degrees (ranging between 3.17–21.95 degrees) with a mean LV twist of 13.81 ± 3.68 degrees. Four subjects showed a cw form of LV-RBR with LV basal rotation (−3.63 ± 1.75 degrees, ranging between −1.85 and −6.12 degrees) and LV apical rotation (−1.20 ± 0.70 degrees, ranging between 0 and −1.71 degrees). Another five cases had a ccw form of LV-RBR with LV basal rotation (0.74 ± 0.34 degrees, ranging between 0 and 1.74 degrees) and LV apical rotation (6.81 ± 3.06 degrees, ranging between 3.22 and 11.94 degrees).

3DSTE-derived LA volumes and volume-based functional properties. When LV-RBR subjects were compared to cases with normally directed LV rotational mechanics, all LA volumes were increased with preserved LA-SVs and (non-significantly) reduced LA-EFs. When subgroups were compared with each other, it has been clarified that an enlargement of the LA with increased volumes was limited only to ccwLV-RBR cases with reduced LA-TAEF, LA-PASV, and LA-PAEF, while cwLV-RBR subjects did not have such LA volumetric abnormalities (Table 2).

3DSTE-derived peak LA strains. Reduced global and mean segmental peak LA-LS could be detected in the group of LV-RBR subjects, which was limited to cases with the ccw form of LV-RBR with the preservation of peak LA-LS in cwLV-RBR cases. Moreover, a concomitant reduction in the mean segmental peak LA-AS was also present in the group of LV-RBR subjects as compared to cases with normally directed LV rotational mechanics. The global peak LA-RS was increased in subjects with cwLV-RBR. When the regionality of peak LA strains was examined, increased basal LA-RS and concomitant LA-3DS with a reduction in midatrial LA-LS and concomitant LA-AS were present in the group of LV-RBR subjects. Comparing ccw and cw LV-RBR cases, superior LA-RS differed significantly. Basal and midatrial LA-LS and midatrial LA-AS were reduced in ccwLV-RBR cases, while superior LA-CS was deteriorated in cwLV-RBR subjects (Table 3 and Table 4).

3DSTE-derived LA strains at atrial contraction. Increased global LA-RS at atrial contraction could be detected in the group of LV-RBR subjects, which was present in both ccw and cw LV-RBR cases. Only in cwLV-RBR subjects was an increased global LA-LS also present. There was a significant difference when comparing the global LA-CS of subjects with ccw vs. cw LV-RBR. When the regionality of LA strains at atrial contraction was examined, basal LA-RS and concomitant LA-3DS proved to be increased. ccwLV-RBR cases had increased basal LA-3DS. Comparing ccw and cw LV-RBR cases, midatrial LA-LS and concomitant LA-AS differed significantly (Table 5 and Table 6).

## 4. Discussion

Owing to the technical developments that have taken place in recent decades, several non-invasive imaging methods suitable for the detailed evaluation of the heart chambers are now available. 3DSTE seems to be optimal for this purpose, and volumetric and functional analysis of LV and LA could be performed at the same time using virtual 3D casts of the heart chambers [11,12,13,14].

The myocardial architecture of the LV is special. Briefly, subepicardial LV fibers run in left-handed direction; in the middle layer, the fibers run circumferentially, while subendocardially, they run in a right-handed direction. As a result, subepicardial and subendocardial LV contractions result in LV rotations occurring in opposite directions. However, due to larger subepicardial rotational radius, a consequentially greater torque is present, and therefore, rotation of the LV subepicardium is expressed. In systole, together with apical counterclockwise LV rotation and basal clockwise LV rotation, the walls of the LV radially thicken (represented by RS with a positive sign); and longitudinally, those of the LV shorten, and circumferentially, they narrow (represented by LS and CS with a negative sign, respectively), resulting in the smallest LV volume. In diastole, the movements of the walls of the LV occur in opposite direction compared to the above described patterns; therefore, its volume is the largest at end-diastole [2,3,4,5,18,19,20].

In systole, LA behaves like a reservoir; its volume is the largest in this phase of the cardiac cycle, while its walls reduce in thickness radially (represented by RS with a negative sign), lengthen longitudinally, and widen circumferentially (represented by LS and CS with a positive sign, respectively). In early diastole, LA is a conduit allowing the blood flowing from the veins to the LV via the open mitral valve, while in late diastole, LA works as a booster pump contracting in order to optimize its emptying. Besides LA strains, all these phases of the LA function can be characterized by volume-based SVs and EFs as well [16,17,21,22].

Although it is known, based on the results of physiological studies, that the LV rotational mechanism is responsible for approx. 40% of ejection, in some cases, it is missing [2,6]. In these cases, the phenomenon that apical and basal regions of the LV that rotate in the same cw or ccw direction are called LV-RBR [6]. Mostly patients with certain pathologies like non-compaction cardiomyopathy, cardiac amyloidosis, acromegaly, and congenital heart disease are affected; however, regarding to the results of recent studies, LV-RBR is present in approx. 5-6% of healthy subjects [6,7]. The present study aimed to understand whether LA volumetric or strain abnormalities could be found in healthy adults showing different kinds of LV-RBR.

According to the findings of the present study, it could be stated that significant LA volumetric and functional abnormalities suggesting LA remodeling are present in healthy adults showing LV-RBR with significant differences between cw and ccw LV-RBR forms. LA volumes are increased only in the ccw form of LV-RBR with reduced LA-TAEF and LA-PAEF, decreased end-systolic global LA-LS, and increased global LA-RS at atrial contraction. In cwLV-RBR, volumetric LA abnormalities are not present, but significantly increased global LA-RS could be detected in both systole and late diastole. Several other regional differences in LA strains could be demonstrated between subjects with the cw and ccw forms of LV-RBR.

A question therefore arises: what is the reason for these abnormalities? In ccwLV-RBR, basal LV rotation occurs in the opposite direction, being pathologically counterclockwise-oriented, even slightly hindering optimal LV filling, which could partially explain the findings detailed above. In cwLV-RBR, when normally counterclockwise LV apical rotation is clockwise oriented, similarly significant abnormalities could not be detected. However, increased global LA-RS at atrial contraction in both ccw and cw LV-RBR could be considered as a compensatory effect. More physiologic studies are warranted to confirm the presented findings even in healthy subjects.

## 5. Limitation

Several limitations could affect these findings:

- Although the participants considered themselves to be healthy, it could not be ruled out with 100% certainty that there was a hidden condition that could have influenced the findings.
- Only a limited number of healthy subjects were examined and compared. However, this is the first study in which group of healthy subjects was examined for such comparisons regarding to LV rotational mechanics. It is true that a larger number of cases would have facilitated a better and statistically stronger analysis.
- Normal reference values of LV rotational parameters showed some age and gender dependency, which could have affected the findings [7].
- The best-known limitation of 3DSTE is its poor image quality, and that of 2D echocardiograpy is still better. The image quality could be significantly affected by the size of the transducer, by the fact that several cardiac cycles are required to create a full volume, by motion and respiratory artifacts, etc.
- 3DSTE is known to be suitable for the measurement of several other parameters, but these were not considered to be the subject of this study.
- The analysis of other heart chambers including the right heart was not aimed to be performed in this study.
- Moreover, already validated LA/LV volumes and strains were not intended to be validated again.
- There can be a debate regarding which atrium/ventricle the atrial and ventricular septum is part of, and it was considered to be part of the LA and the LV during the analysis.
- Only a limited number of healthy individuals presented LV-RBR in this study. According to the literature data, the ratio of healthy individuals presenting LV-RBR in a healthy population is not exactly determined.

## 6. Conclusions

In healthy adults presenting LV-RBR, subclinical LA remodeling could be detected in both forms of LV-RBR, but more pronounced in those who present a counterclockwise-oriented form.

## Figures and Tables

**Table 1:** Demographic data of healthy subjects.

	All Healthy Subjects (*n* = 165)	Normally Directed LV Rotational Mechanics (*n* = 156)	All LV-RBR (*n* = 9)
male gender (%)	75 (45)	72 (46)	3 (33)
mean age (years)	33.1 ± 12.3	32.7 ± 11.9	34.7 ± 10.8
body mass index (kg/m^2^)	24.0 ± 1.9	24.2 ± 1.7	23.7 ± 1.9
body surface area (m^2^)	1.82 ± 0.14	1.81 ± 0.17	1.83 ± 0.16
systolic blood pressure (mm Hg)	122.4 ± 4.1	123.0 ± 4.3	122.1 ± 3.8
diastolic blood pressure (mm Hg)	72.9 ± 4.2	73.4 ± 4.4	72.6 ± 4.0
heart rate (1/s)	71.7 ± 2.9	72.4 ± 3.0	71.3 ± 2.8
LA (mm)	36.7 ± 4.0	36.7 ± 4.0	35.9 ± 3.9
LV-EDD (mm)	48.1 ± 3.7	48.1 ± 3.7	48.0 ± 3.9
LV-EDV (mL)	106.8 ± 22.9	106.6 ± 22.9	110.2 ± 22.7
LV-ESD (mm)	32.0 ± 3.3	31.9 ± 3.2	33.0 ± 4.8
LV-ESV (mL)	36.6 ± 9.3	36.3 ± 9.0	40.3 ± 12.5
IVS (mm)	8.9 ± 1.5	8.9 ± 1.6	9.5 ± 1.3
LV-PW (mm)	9.1 ± 1.6	9.0 ± 1.7	9.8 ± 1.0
LV-EF (%)	65.8 ± 4.9	65.9 ± 4.9	64.2 ± 5.7
E (cm/s)	79.3 ± 17.2	79.6 ± 17.1	74.3 ± 17.7
A (cm/s)	64.8 ± 20.0	65.5 ± 20.2	54.0 ± 10.4

Data are presented as mean ± standard deviation format. LV = left ventricular; RBR = rigid body rotation. LA = left atrium; LV = left ventricle; EDD = end-diastolic diameter; EDV = end-diastolic volume; ESD = end-systolic diameter; ESV = end-systolic volume; IVS = interventricular septum; PW = posterior wall; EF = ejection fraction; E and A = early and late diastolic mitral inflow velocities.

**Table 2:** Three-dimensional speckle-tracking echocardiography-derived left atrial volumes and volume-based functional properties of healthy subjects showing normally directed left ventricular rotational mechanics versus left ventricular ‘rigid body rotation’.

	All Healthy Subjects (*n* = 165)	Normally Directed LV Rotational Mechanics (*n* = 156)	All LV-RBR (*n* = 9)	ccwLV-RBR (*n* = 5)	cwLV-RBR (*n* = 4)
LA-V_max_ (mL)	40.9 ± 13.1	40.4 ± 12.8	48.7 ± 15.5 *	56.5 ± 16.7 *	39.0 ± 4.4
LA-V_preA_ (mL)	27.7 ± 11.8	27.2 ± 11.2	36.5 ± 17.1 *	46.5 ± 16.7 *	24.0 ± 5.7 †
LA-V_min_ (mL)	19.4 ± 8.2	19.0 ± 7.6	26.9 ± 12.2 *	34.3 ± 13.5 *	17.8 ± 4.0 †
LA-TASV (mL)	21.5 ± 8.2	21.5 ± 8.3	21.8 ± 5.6	22.2 ± 6.7	21.2 ± 3.8
LA-TAEF (%)	52.7 ± 11.9	53.0 ± 11.9	46.5 ± 10.7	40.1 ± 8.0 *	54.5 ± 7.9 †
LA-PASV (mL)	13.2 ± 5.6	13.2 ± 5.7	12.2 ± 3.9	10.0 ± 1.8 *	15.0 ± 3.9 †
LA-PAEF (%)	33.3 ± 12.6	33.6 ± 12.5	28.1 ± 13.2	19.4 ± 7.7 *	38.8 ± 10.6 †
LA-AASV (mL)	8.3 ± 5.8	8.2 ± 5.8	9.6 ± 5.7	12.2 ± 6.3	6.3 ± 2.2
LA-AAEF (%)	28.9 ± 11.9	29.1 ± 12.1	25.6 ± 6.2	25.7 ± 6.0	25.5 ± 6.5

Data are presented as mean ± standard deviation format. * *p* < 0.05 vs. normally directed LV rotational mechanics, † *p* < 0.05 vs. ccwLV-RBR. cw = clockwise; ccw = counterclockwise; LV = left ventricular; RBR = rigid body rotation; LA = left atrial; V_max_ = maximum LA volume; V_preA_ = pre-atrial contraction LA volume; V_min_ = minimum LA volume; TASV = total LA stroke volume; PASV = passive LA stroke volume; AASV = active LA stroke volume; TAEF = total LA emptying fraction; PAEF = passive LA emptying fraction; AAEF = active LA emptying fraction.

**Table 3:** Three-dimensional speckle-tracking echocardiography-derived peak global and mean segmental left atrial strains of healthy subjects showing normally directed left ventricular rotational mechanics versus left ventricular ‘rigid body rotation’.

	All Healthy Subjects (*n* = 165)	Normally Directed LV Rotational Mechanics (*n* = 156)	All LV-RBR (*n* = 9)	ccwLV-RBR (*n* = 5)	cwLV-RBR (*n* = 4)
LA-GRS (%)	−14.9 ± 8.1	−14.7 ± 8.0	−17.3 ± 9.0	−12.7 ± 7.9	−23.4 ± 6.3 *
LA-GCS (%)	33.6 ± 15.3	33.9 ± 15.6	27.3 ± 7.7	28.0 ± 8.6	26.5 ± 6.4
LA-GLS (%)	26.3 ± 9.1	26.6 ± 9.0	19.8 ± 8.0 *	15.1 ± 4.7 *	25.6 ± 7.4 †
LA-G3DS (%)	−7.5 ± 5.9	−7.4 ± 5.7	−9.0 ± 8.4	−6.3 ± 6.6	−12.3 ± 9.3
LA-GAS (%)	67.5 ± 28.1	68.4 ± 28.4	51.5 ± 14.7	47.0 ± 14.8	57.1 ± 12.4
LA-msRS (%)	−19.1 ± 6.9	−19.1 ± 6.9	−20.2 ± 6.3	−18.1 ± 7.6	−22.8 ± 2.4
LA-msCS (%)	37.6 ± 14.4	38.0 ± 14.6	30.1 ± 7.6	31.9 ± 7.0	27.9 ± 7.7
LA-msLS (%)	29.3 ± 8.3	29.8 ± 8.1	21.1 ± 6.5 *	18.0 ± 4.1 *	25.1 ± 6.8
LA-ms3DS (%)	−12.5 ± 5.0	−12.5 ± 5.0	−12.9 ± 5.5	−12.6 ± 6.3	−13.3 ± 4.1
LA-msAS (%)	73.1 ± 26.9	74.2 ± 27.0	53.8 ± 14.8 *	52.6 ± 13.2	55.2 ± 16.6

Data are presented as mean ± standard deviation format. * *p* < 0.05 vs. normally directed LV rotational mechanics, † *p* < 0.05 vs. ccwLV-RBR. cw = clockwise; ccw = counterclockwise; LV = left ventricular; RBR = rigid body rotation; LA = left atrial; GRS = global radial strain; GCS = global circumferential strain; GLS = global longitudinal strain; G3DS = global three-dimensional strain; GAS = global area strain; msRS = mean segmental radial strain; msCS = mean segmental circumferential strain; msLS = mean segmental longitudinal strain; ms3DS = mean segmental three-dimensional strain; msAS = mean segmental area strain.

**Table 4:** Three-dimensional speckle-tracking echocardiography-derived regional peak left atrial strains of healthy subjects showing normally directed left ventricular rotational mechanics versus left ventricular ‘rigid body rotation’.

	All Healthy Subjects (*n* = 165)	Normally Directed LV Rotational Mechanics (*n* = 156)	All LV-RBR (*n* = 9)	ccwLV-RBR (*n* = 5)	cwLV-RBR (*n* = 4)
Basal LA-RS (%)	−17.8 ± 9.0	−17.5 ± 8.9	−23.6 ± 8.8 *	−22.0 ± 9.5	−25.7 ± 7.3
Midatrial LA-RS (%)	−18.9 ± 7.6	−18.8 ± 7.4	−20.5 ± 9.2	−17.0 ± 9.0	−24.8 ± 7.4
Superior LA-RS (%)	−21.7 ± 11.8	−21.8 ± 11.9	−19.9 ± 10.2	−13.8 ± 7.6	−27.6 ± 7.3 †
Basal LA-CS (%)	41.8 ± 15.2	42.1 ± 15.5	38.2 ± 7.4	37.1 ± 7.5	39.6 ± 7.1
Midatrial LA-CS (%)	32.2 ± 12.8	32.5 ± 12.9	26.3 ± 8.6	22.3 ± 7.5	31.2 ± 7.1
Superior LA-CS (%)	39.4 ± 26.1	40.1 ± 26.4	27.9 ± 17.3	38.5 ± 15.3	14.6 ± 8.0 *†
Basal LA-LS (%)	22.7 ± 11.0	22.9 ± 11.0	19.5 ± 9.8	12.7 ± 1.5 *	27.9 ± 9.3 †
Midatrial LA-LS (%)	37.1 ± 12.5	37.6 ± 12.3	27.7 ± 13.1 *	20.1 ± 6.9 *	37.3 ± 12.7
Superior LA-LS (%)	22.7 ± 14.7	27.9 ± 14.7	19.1 ± 10.8	22.6 ± 13.5	14.7 ± 2.0
Basal LA-3DS (%)	−12.5 ± 7.3	−12.2 ± 7.1	−17.3 ± 8.8 *	−16.5 ± 7.9	−18.2 ± 9.7
Midatrial LA-3DS (%)	−11.5 ± 5.6	−11.5 ± 5.4	−11.4 ± 8.4	−11.3 ± 7.7	−11.5 ± 9.1
Superior LA-3DS (%)	−14.1 ± 8.5	−14.3 ± 8.5	−11.6 ± 7.0	−8.3 ± 4.9	−15.7 ± 7.2
Basal LA-AS (%)	63.9 ± 24.7	64.2 ± 24.9	58.6 ± 20.2	49.2 ± 7.4	70.4 ± 24.5
Midatrial LA-AS (%)	75.7 ± 27.5	76.8 ± 27.3	56.0 ± 21.8 *	42.3 ± 12.8 *	73.1 ± 18.5 †
Superior LA-AS (%)	83.7 ± 62.4	85.3 ± 63.3	56.2 ± 33.4	73.0 ± 33.5	35.3 ± 17.6

Data are presented as mean ± standard deviation format. * *p* < 0.05 vs. normally directed LV rotational mechanics, † *p* < 0.05 vs. ccwLV-RBR. cw = clockwise; ccw = counterclockwise; LV = left ventricular; RBR = rigid body rotation; LA = left atrial; RS = radial strain; CS = circumferential strain; LS = longitudinal strain; 3DS = three-dimensional strain; AS = area strain.

**Table 5:** Three-dimensional speckle-tracking echocardiography-derived global and mean segmental left atrial strains at atrial contraction of healthy subjects showing normally directed left ventricular rotational mechanics versus left ventricular ‘rigid body rotation’.

	All Healthy Subjects (*n* = 165)	Normally Directed LV Rotational Mechanics (*n* = 156)	All LV-RBR (*n* = 9)	ccwLV-RBR (*n* = 5)	cwLV-RBR (*n* = 4)
LA-GRS (%)	−5.4 ± 5.5	−5.2 ± 5.2	−9.9 ± 7.1 *	−9.7 ± 6.1 *	−10.0 ± 8.2 *
LA-GCS (%)	13.6 ± 9.6	13.6 ± 9.7	12.9 ± 8.7	18.3 ± 5.4	6.0 ± 7.2 †
LA-GLS (%)	8.4 ± 7.4	8.3 ± 7.3	10.2 ± 8.3	5.9 ± 3.5	15.7 ± 9.2 *
LA-G3DS (%)	−3.1 ± 4.5	−3.1 ± 4.5	−3.8 ± 4.4	−4.7 ± 5.2	−2.7 ± 2.7
LA-GAS (%)	22.9 ± 17.4	23.1 ± 17.6	20.5 ± 13.7	25.6 ± 11.2	14.2 ± 13.9
LA-msRS (%)	−7.9 ± 4.3	−7.8 ± 4.2	−9.5 ± 4.4	−9.3 ± 5.1	−9.8 ± 3.3
LA-msCS (%)	15.3 ± 8.3	15.3 ± 8.5	14.7 ± 5.6	17.2 ± 5.3	11.5 ± 4.0
LA-msLS (%)	9.9 ± 5.2	9.9 ± 5.2	9.6 ± 4.3	7.4 ± 3.7	12.4 ± 3.2
LA-ms3DS (%)	−5.1 ± 3.8	−5.0 ± 3.8	−6.1 ± 3.9	−5.9 ± 4.8	−6.4 ± 2.2
LA-msAS (%)	26.2 ± 14.5	26.2 ± 14.7	25.9 ± 9.6	26.9 ± 10.7	24.8 ± 7.7

Data are presented as mean ± standard deviation format. * *p* < 0.05 vs. normally directed LV rotational mechanics, † *p* < 0.05 vs. ccwLV-RBR. cw = clockwise; ccw = counterclockwise; LV = left ventricular; RBR = rigid body rotation; LA = left atrial; GRS = global radial strain; GCS = global circumferential strain; GLS = global longitudinal strain; G3DS = global three-dimensional strain; GAS = global area strain; msRS = mean segmental radial strain; msCS = mean segmental circumferential strain; msLS = mean segmental longitudinal strain; ms3DS = mean segmental three-dimensional strain. msAS = mean segmental area strain.

**Table 6:** Three-dimensional speckle-tracking echocardiography-derived regional left atrial strains at atrial contraction of healthy subjects showing normally directed left ventricular rotational mechanics versus left ventricular ‘rigid body rotation’.

	All Healthy (*n* = 165)	Normally Directed LV Rotational Mechanics (*n* = 156)	All LV-RBR (*n* = 9)	ccwLV-RBR (*n* = 5)	cwLV-RBR (*n* = 4)
Basal LA-RS (%)	−7.5 ± 5.4	−7.2 ± 5.4	−11.8 ± 5.1 *	−11.4 ± 5.1	−12.2 ± 4.9
Midatrial LA-RS (%)	−7.6 ± 4.6	−7.6 ± 4.6	−8.4 ± 4.8	−8.7 ± 5.7	−8.0 ± 3.3
Superior LA-RS (%)	−8.9 ± 9.9	−9.0 ± 8.0	−7.9 ± 6.2	−7.0 ± 7.4	−9.1 ± 4.0
Basal LA-CS (%)	17.0 ± 9.2	17.0 ± 9.4	16.9 ± 4.9	19.1 ± 4.5	14.3 ± 3.9
Midatrial LA-CS (%)	13.0 ± 8.3	13.1 ± 8.5	12.2 ± 4.4	12.1 ± 4.9	12.3 ± 3.7
Superior LA-CS (%)	15.7 ± 14.4	15.8 ± 14.4	15.5 ± 14.5	30.2 ± 24.9	6.9 ± 7.7
Basal LA-LS (%)	7.6 ± 5.2	7.7 ± 5.3	6.1 ± 3.2	5.9 ± 2.6	6.5 ± 3.8
Midatrial LA-LS (%)	11.2 ± 7.7	11.1 ± 7.6	12.9 ± 8.0	7.2 ± 3.2	20.0 ± 6.3 *†
Superior LA-LS (%)	11.0 ± 8.3	11.0 ± 8.2	11.2 ± 8.6	10.9 ± 3.4	11.5 ± 7.5
Basal LA-3DS (%)	−5.0 ± 5.1	−4.8 ± 5.0	−8.6 ± 4.7 *	−9.5 ± 5.5 *	−7.4 ± 3.2
Midatrial LA-3DS (%)	−4.7 ± 4.2	−4.6 ± 4.1	−5.0 ± 5.3	−5.6 ± 6.7	−4.3 ± 2.3
Superior LA-3DS (%)	−5.9 ± 6.7	−6.0 ± 6.7	−4.1 ± 6.8	−1.1 ± 6.9	−7.8 ± 4.2
Basal LA-AS (%)	23.6 ± 13.5	23.6 ± 13.8	23.5 ± 5.8	25.0 ± 6.4	21.7 ± 4.4
Midatrial LA-AS (%)	26.0 ± 15.6	26.1 ± 15.8	25.0 ± 11.5	18.1 ± 7.9	33.7 ± 9.4 †
Superior LA-AS (%)	30.2 ± 29.1	30.2 ± 29.2	30.7 ± 28.6	42.4 ± 30.5	16.1 ± 16.9

Data are presented as mean ± standard deviation format. * *p* < 0.05 vs. normally directed LV rotational mechanics, † *p* < 0.05 vs. ccwLV-RBR. cw = clockwise; ccw = counterclockwise; LV = left ventricular; RBR = rigid body rotation; LA = left atrial; RS = radial strain; CS = circumferential strain; LS = longitudinal strain; 3DS = three-dimensional strain; AS = area strain.

## Data Availability

The original contributions presented in the study are included in the article, further inquiries can be directed to the corresponding author.
